# Supplementary figures and images for: Translesion synthesis polymerases are dispensable for C. elegans reproduction but suppress genome scarring by polymerase theta-mediated end joining
Source: PLoS Genet. 2020 Apr 24;16(4):e1008759. doi: 10.1371/journal.pgen.1008759 (PMC7202663; doi:10.1371/journal.pgen.1008759)

Supplemental Figure 1

A

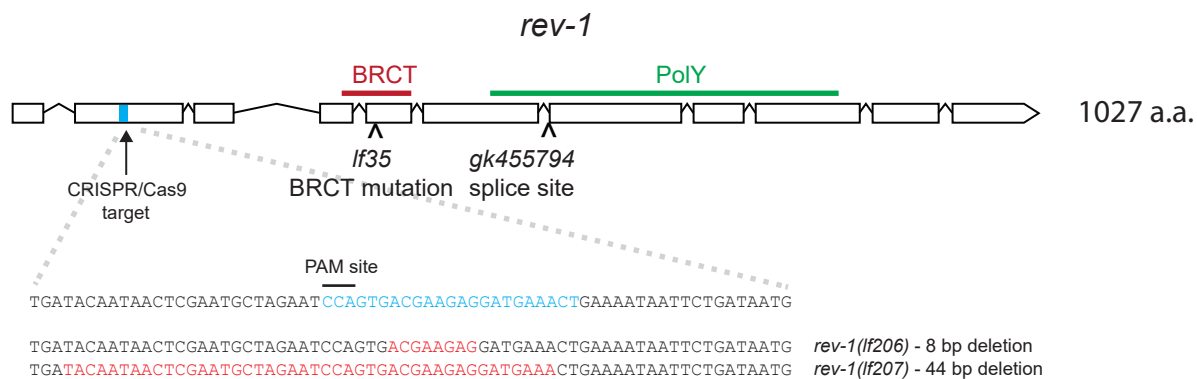

B

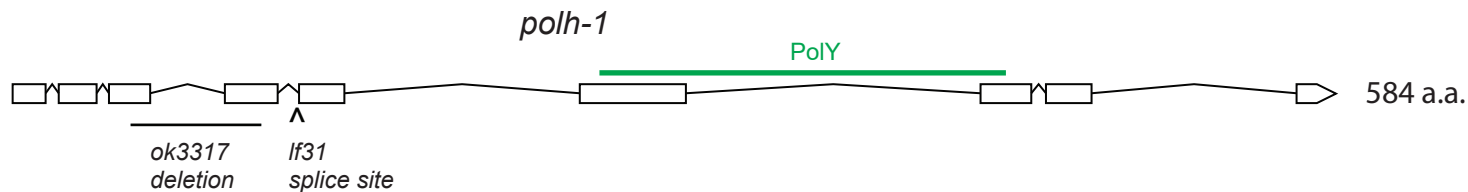

C

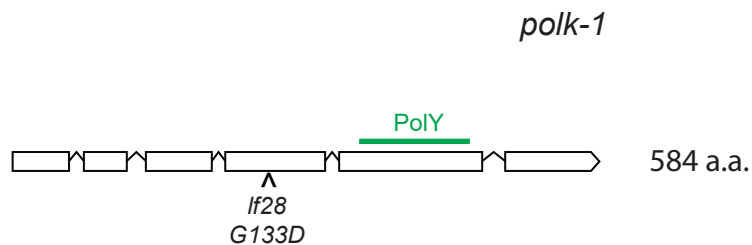

D

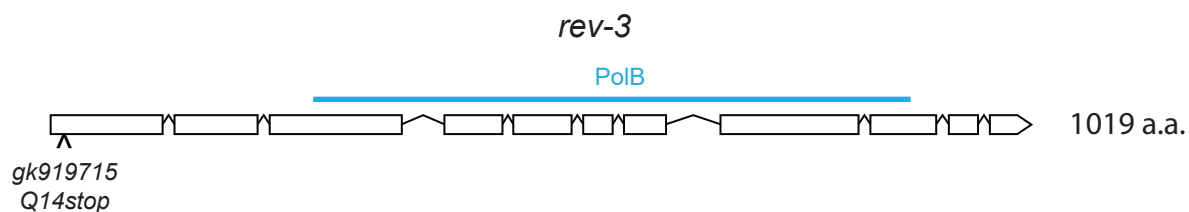

Supplement: S1 Fig — A) rev-1, B) polh-1, C) polk-1 and D) rev-3. (PDF) [file pgen.1008759.s001.pdf]

Supplemental Figure 2

*dog-1(gk10)*

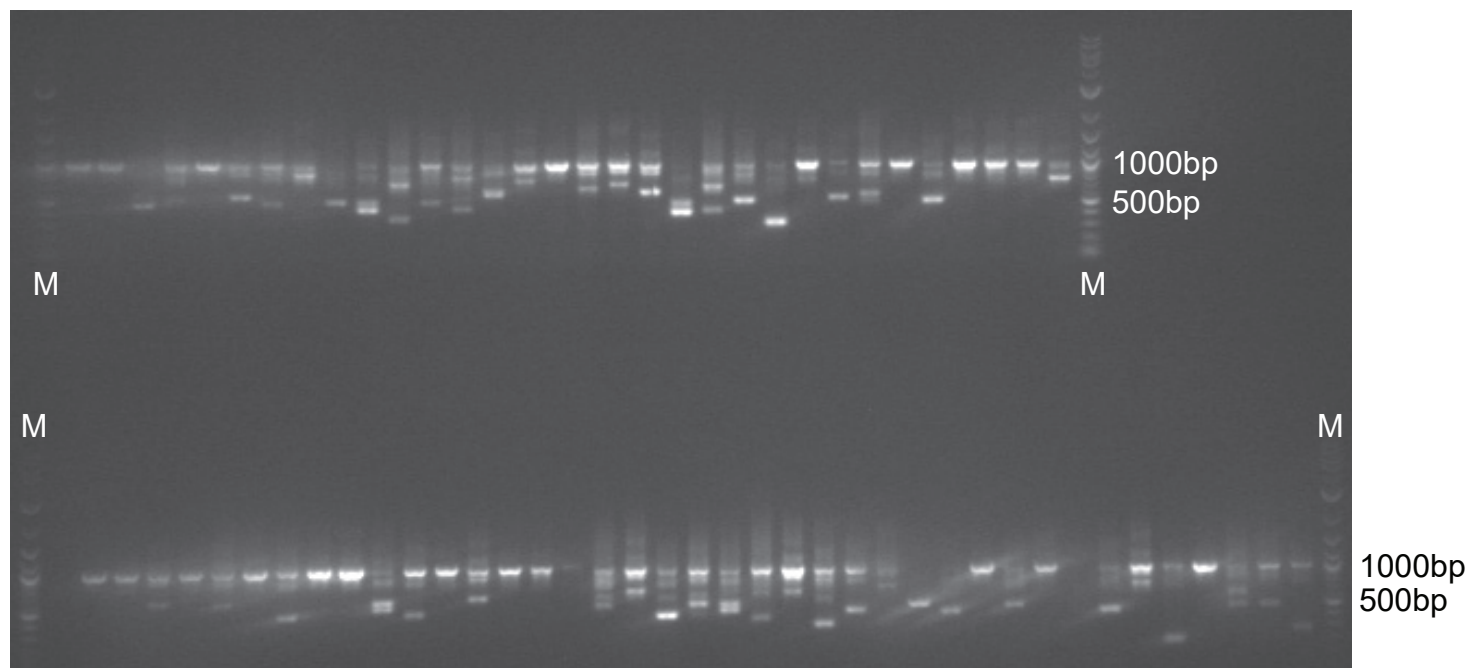

50/69 wells display a G4 deletion

*dog-1(gk10) rev-1(gk455794)*

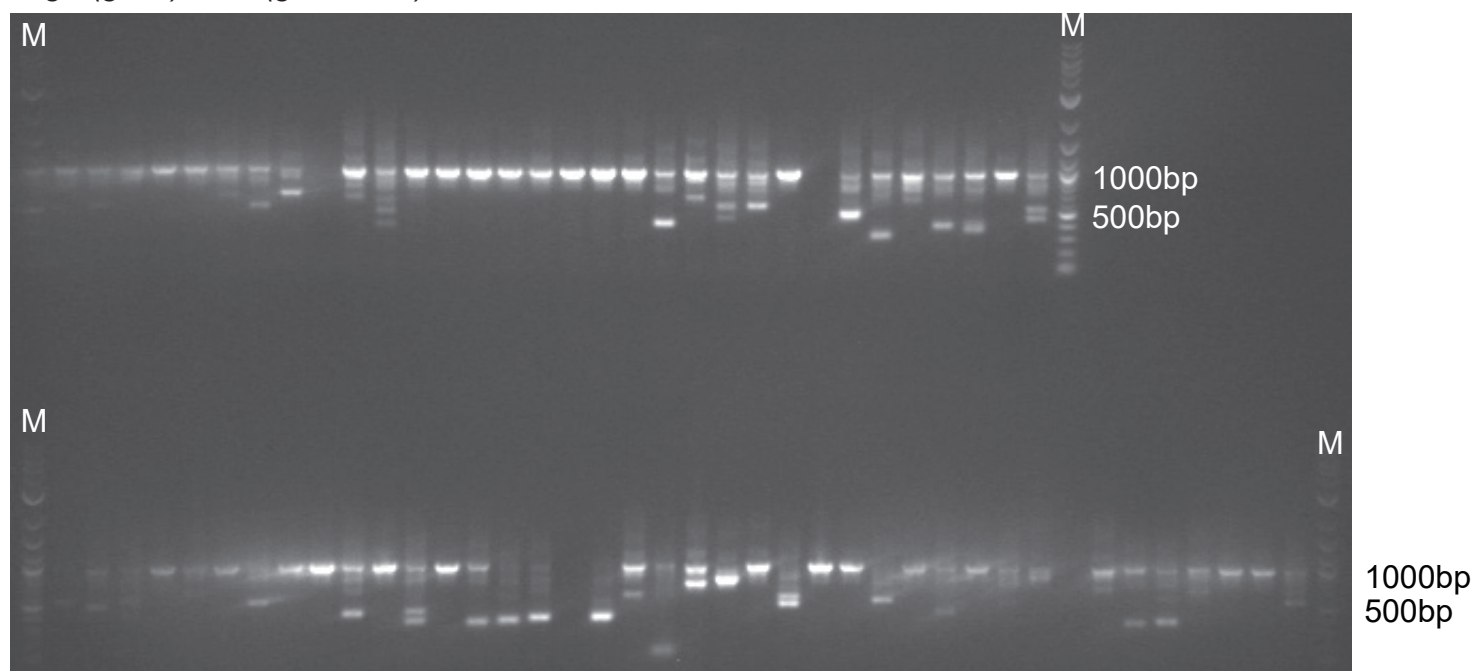

36/69 wells display a G4 deletion

Supplement: S2 Fig — Nested PCR was performed on genomic G4 site qua1466 (expected size: 1000bp). 5 adult worms per well lysed in 15 ul lysis buffer. 1ul lysis per well was used as a template for a nested PCR. No additional genomic instability was observed in dog-1 deficient animals after loss of REV-1. M denotes DNA marker. Wells were scored as positive when one or more bands appeared <1000bp. Empty wells were not included in the quantification. (PDF) [file pgen.1008759.s002.pdf]

A

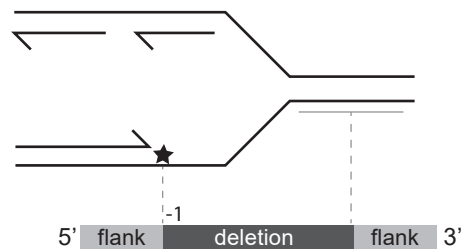

B

*polh-1 polk-1*  
deletions ( $n = 163$ )

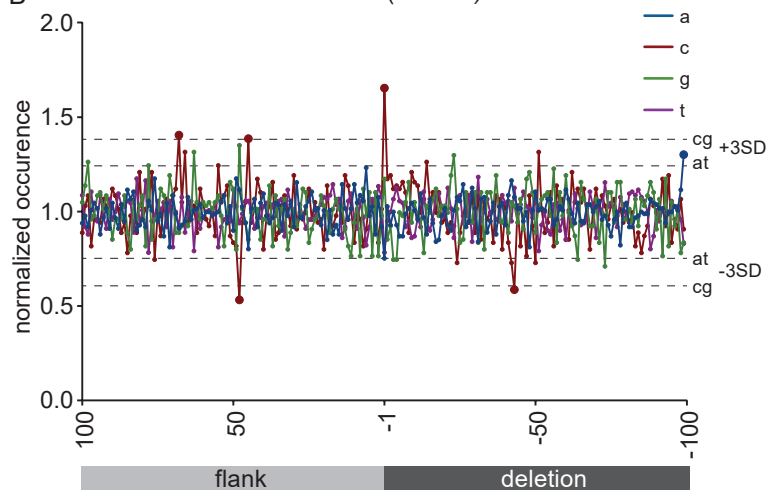

C

*polh-1 polk-1 rev-1*  
deletions ( $n = 208$ )

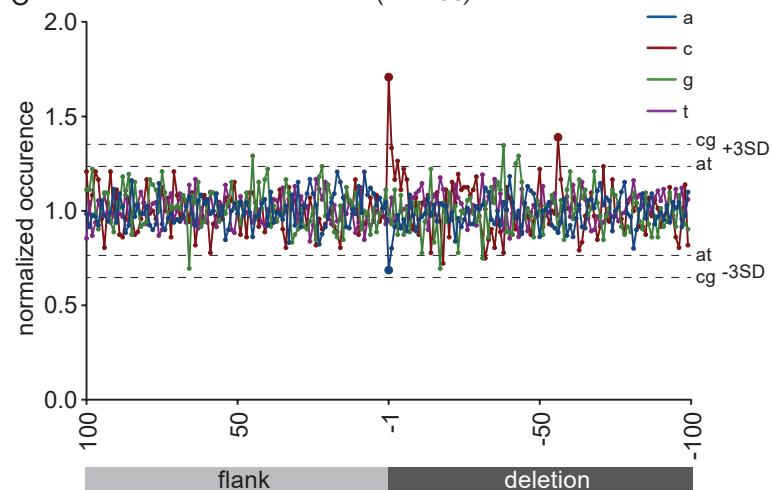

D

*polh-1 polk-1*  
delins ( $n = 40$ )

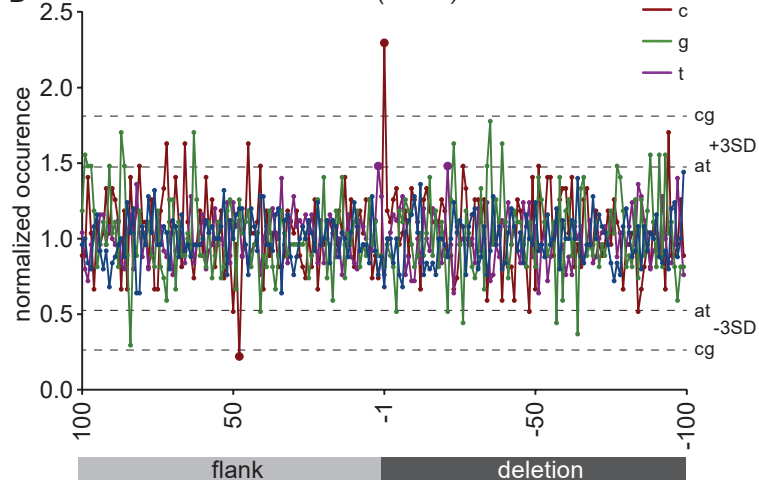

E

*polh-1 polk-1 rev-1*  
delins ( $n = 58$ )

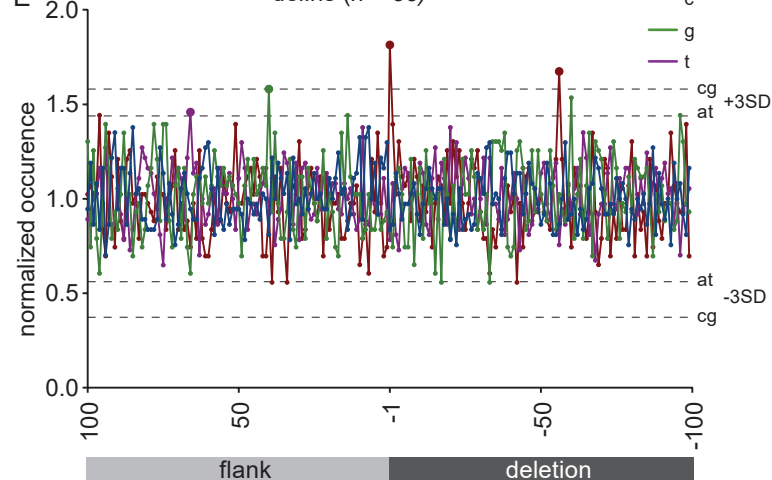

Supplement: S3 Fig — A) Schematic illustration of the concept that one junction of DNA-damage-induced deletions is defined by the nascent strand blocked at sites of DNA damage. In this hypothesis, the replication-blocking lesion may dictate position -1, being the outermost nucleotide of the lost sequence. B-C) The base composition of all deletion breakpoints, normalized to the relative AT/CG content around the breakpoints (from +100 to -100) in polh-1 polk-1 (B) and polh-1 polk-1 rev-1 (C). Position 100 to 1 reflects the sequence that is retained in the deletion alleles; position -1 to -100 reflects the sequence that is lost. Dashed lines represent three times the SD. Data points outside these boundaries are marked with an enlarged dot. D) as in B, but now only for deletions with insertions. E) as in C, but now only for deletions with insertions. (PDF) [file pgen.1008759.s003.pdf]
