# Supplementary material for: Translesion synthesis polymerases are dispensable for C. elegans reproduction but suppress genome scarring by polymerase theta-mediated end joining
Source: PLoS Genet. 2020 Apr 24;16(4):e1008759. doi: 10.1371/journal.pgen.1008759 (PMC7202663; doi:10.1371/journal.pgen.1008759)
Supplement: Attachment — Submitted filename: Response to reviewers.docx [file pgen.1008759.s006.docx]

**Point to point response PGENETICS-D-20-00260**

We would like to thank all reviewers for their time and support, and for the constructive remarks and suggestions, which in our opinion helped us to increase the quality and readability of our manuscript.

**Reviewer #1:**

Specific comments:
1. It would be helpful if the authors could summarize their analyses of mutations per generation in a table that would also include literature data as comparison as some of the single TLS mutants are referred to in the literature and not analyzed again here.

This is now included in the manuscript as supplementary table 1

2. SD should be used instead of SEM whenever distinct populations (e.g. mutant vs. wildtype) are compared.

This has now been corrected for the relevant figures. Figure legends for figure 1B mistakenly stated SEM, but SD was already shown here.

**Reviewer #2:**

Further comments:

Introduction: When listing the TLS polymerases, please also make a mention of the TLS role of pol delta, especially PolD3 (see e.g. PMID: 25628356).

This is now mentioned with the inclusion of the suggested reference as an example.

p6 Fig. 1F unc-93 reversion assay: again, it would be great to have some more sequences. The authors should also analyse the context of the deletions, specifying if they show microhomology.

We would also have preferred larger numbers, but it should be noted that getting to the numbers presented has already been a substantial amount of work given the very low spontaneous mutation frequencies in both wild type and mutant. The presence or absence of micro-homology at the deletion junction has now been indicated (given the low numbers, it however did not induce conclusive remarks)

p9: The polh polk rev1 triple mutant had marginally fewer mutations than the polh polk double mutant, indicating a weak role for rev1 in this background. But considering that a major function of rev1 is the recruitment of other Y family polymerases rather than DNA synthesis, this could also be an epistatic relationship. The recruitment role of rev1 is discussed in the next section, but only with respect to pol zeta. It is possible that rev1 is needed for both polh/polk recruitment and polz recruitment, and both of these are needed for lesion bypass according to the two-polymerase model.

If REV1 is needed for recruitment of the other Y-family polymerase, we would have expected that the number of deletions in the genomes of *rev-1* mutant animals are comparable to those in polh-1 polk-1 mutant animals, which it is not. A similar argument holds true for recruiting all other TLS polymerases.

Alternatively, the explanation for the much stronger phenotype of the polh polk double mutant compared to rev1 or rev1 rev3 could suggest that polh and polk are primarily recruited through a rev1-independent mechanism, presumably PCNA ubiquitylation. This mechanism has been investigated recently (PMID: 31450086) and should be discussed.

This possibility as well as the reference is now included in the main text.

In the discussion, please further emphasise that the results are C. elegans specific, and compare and contrast them with yeast and vertebrate data where available.

We now stated at the start of the discussion “Here, we have shown in *C. elegans* that lack of TLS polymerases leads to an increase in the overall mutational load when animals are grown under unperturbed conditions, implying that TLS action suppresses mutagenesis.” to emphasize the biological context. We now also added “in *C. elegans*” in our final conclusive (general) statement on the role of TLS in genome protection. A comparison to yeast mutagenesis data is troubled by the fact that yeast does not encode polymerase theta, hence the downstream consequence of TLS loss will be different but not necessarily because of different TLS biology (and we wished to stay away from a lengthy discussion on TMEJ).

Please compare the spectrum of deletions in the TLS mutants to the recently published COSMIC version 3 indel signatures.

We now refer to the similarity of our data to COSMIC ID6, but only with respect to micro-homology as we have no information on the size of those deletions (these are described as >5) nor whether they map to specific bases (such as we here found for guanines).

Importantly, there needs to be a supplementary table for the WGS data, showing the precise number of worms/worm populations sequenced, the number of generations, the number and classification of mutations in each sample, and the list of mutations (with chromosomal position) for each sample. I cannot find any of this information in the manuscript. It is not sufficient to share the raw sequence files.

This is now included as Supplemental Table 2.

It would also be useful to show a summary of the mutation data in a simple table in the text. Currently these data are split to numerous graphs, with different scales, making comparisons difficult.
These tables should also include previously published data that was re-analysed here.

Such a table, also requested by Rev#1 is now included as Supplementary Table 1. We also addressed this issue by combining the mutation data in one figure (as suggested by reviewer #3), which aids comparisons.

The doi link of ref 17 is incorrect.

We apologise for the error, and have now corrected this.

**Reviewer #3:**

Overall, the experiments and analyses presented here are largely sound and well documented, and the topic is certainly appropriate for PLoS Genetics. However, the work is substantially weakened by statements made throughout the manuscript that conflate actual experimental findings with interpretations. That is, the authors claim to have demonstrated Y, when in fact they have actually demonstrated X, and Y is the favored interpretation. This failure to make rigorous distinctions between: a) experimental results and b) interpretations and conclusions based on the results is problematic for several reasons. It is misleading to more naïve readers who may not recognize the conflation, and it is also misleading to busy expert readers who may assume that a different type of assay must have been performed in order to reach the stated conclusion. For example, the authors have a tendency to make statements that would appear to require biochemistry to support them. The authors must be careful to articulate their conclusions in a manner that aligns with the actual assays performed and the type of data from which they are derived.

The above-described problem, while pervasive and serious, can be addressed almost entirely by textual revisions. Many instances are flagged here, but please note that this listing is not comprehensive, and the authors should make appropriate revisions throughout the manuscript.

We apologise for being too loose with our language; we had no intention whatsoever to mislead. We have now amended the text of our manuscript to be more precise on a lot of occasions.

Title:
“suppress genome scarring at sites of base damage”
It is true that TLS polymerases (collectively) suppress formation of spontaneous deletions. However, it has not been demonstrated that the inferred breaks that give rise to the deletions occur at sites of base damage.

We have changed the title.

Abstract:
It is shown that the TLS polymerases antagonize the formation of deletions, but the idea that they prevent DSBs from occurring is an indirect inference. And again, the idea that breaks are occurring at sites of base damage is hypothesized based on biochemistry in other systems, but it not demonstrated here; nor is directly demonstrated that guanine adducts are the main source of DNA damage requiring TLS for bypass (see comments below regarding discussion), although the data are consistent with this interpretation. Statements made in the abstract must make a clear distinction between what is shown, and what are (likely) interpretations supported by the data. It is entirely appropriate to interpret data in light of a model, as long as the distinctions are clear.

The abstract text has been rewritten accordingly.

The last sentence seems like an odd closer given the statement just prior that TLS in C. elegans is predominantly error free- can the authors articulate: where is the risk that needs to be outweighed?

Agreed, and we have changed the text.

Results:
P5 l 102-109: 1) cannot equate RAD-51 foci with DSBs; RAD-51 foci do NOT necessarily represent DSBs, as RAD-51 can also associate with other types of lesions, e.g. ss gaps.
2) “apparently, these spontaneous DSBs do not affect proliferation…” Based on the info provided, one cannot assume repair- if lesions such as breaks or extensive ss DNA persisted into meiotic prophase, they would almost certainly be detected by DNA damage checkpoints and eliminated by apoptosis. Note that the authors could potentially test whether apoptosis is elevated in these mutants, but given that it is estimated that >50 of oogenic germ cell nuclei are eliminated during WT meiosis, even if there were a significant increase in nuclei harboring unrepaired DNA lesions, this might not be sufficient to move the needle on apoptosis frequency. The best thing to do here would be to simply remove the problematic narrative and jump into the compelling evidence for altered mutation profiles.

We would like to thank the reviewer for this suggestion. The text has been amended accordingly.

P6, Fig 1:
l 108, state “asked whether mutation induction is elevated”- but while the assay in this section evaluates mutation profile, insufficient information about mutation frequency is provided here. Please elaborate on effects on mutation frequency, or if there are concerns that the unc-93 mutation assay was performed in a way that would not provide reliable information regarding frequency, modify the set-up and other text to focus this section on mutation profile.

Again we would like to thank the reviewer for suggesting alternative solutions to the issue at hand. We have now rewritten the section to omit remarks on frequency as indeed we are not convinced that the *unc-93* assay (as was performed) allows us to be conclusive on mutation rates.

Statistically analysis (e.g. Fisher exact test) evaluating the relative numbers of SNPs vs indels among the unc-93 mutations from WT vs revBRCT mutant indicate that there is not statistical support to conclude that the mutation profiles are different.

We could perhaps make the point of altered mutagenesis in revBRCT stronger by referring to previously published *unc-93* data obtained in wild type and various RNAi knockdown conditions (e.g. Pothof et al., Genes Dev 2003), but decided against it because we felt it would put too much emphasis on this rather smallish observation. Instead, we omitted a conclusive remark about revBRCT.

“From this data we conclude…..bypass of endogenous lesions”: This is NOT a lesion bypass assay, please align conclusion with the assay.

“… role in SUPRESSING spontaneous mutagenesis LIKELY by suppressing the formation…”

The text of the manuscript at these sections have now been changed in order to be precise.

P7 Fig 2
Please provide more information (could be in Figure legend or methods) regarding how mutation rates were deduced from the mutation accumulation lines.

Additional information has now been included in the methods section.

L 145 “…identified 15 cases…” Here, the point scan actually be made more strongly- rather than calling out specifically one deletion size class, the authors can point out that the whole size distribution is very different (2C), a result with very strong statistical support with Mann-Whitney test (p value should be provided). Suggest stating: “There is both an increase in the incidence of deletions and a substantial difference in the size distributions of the deletions observed.”

We recognise that the suggestion is strong but prefer to maintain the original phrasing. It is true that the distribution changes (for which we now provide statistical support) but to us that most likely results from the appearance of one particular class, *i.e.* deletions of >50. Rephrasing as suggested could lead to the idea that a premutagenic lesion gives rise to size A in wild-type and size B in *rev-1* mutants, while we think our data best fit a scenario where size A deletions are not affected by REV-1 status, but particularly size B deletions are resulting from absent TLS. We included statistical support.

L148 To be able to make a statement about a class of mutations being present in one genotype but absent in another, need to provide information about the numbers and to address whether there is statistical support for there being a real difference between genotypes.

We now rephrased the sentence such that it just notes the observation.

I now realise that some of our phrasing leans on a substantial body of work (WGS of other samples) that is not (and cannot be) in the manuscript, leading us to sometimes be too conclusive. In this case, for instance, we have additional WGS data of a large variety of mutants, the majority of which do not have an altered mutation profile. With a certain degree of confidence, we could consider this data in part as surrogate wild-type conditions, making us quite confident that e.g. the enhanced levels of deletions of >50 in the revBRCT or the complex genome rearrangements in the *rev-1* mutants are biologically meaningful. We obviously realise that this is not scientifically correct, and hence did (and will) not include it in our reasoning, but apparently it led us to use too definitive wording.

P8 l 175-178
Here the set-up narrative is flawed- one can conclude that repair of UV-induced lesions in the absence of REV1 is Pol theta dependent, CONSISTENT WITH…(interpretation).

We changed “to substantiate the conclusion” as if it was a fact, not an interpretation, to “to further assess the notion”.

P9 L194. Here, and elsewhere (and possibly in the Discussion), the authors need to consider the possibility that elimination of damage-containing cells via apoptosis could profoundly affect the embryonic survival metric; at a minimum, they need to discuss this issue as it is relevant to the conclusion that TLS polymerases are non-essential.

We have included this biology in the discussion section.

L199-202. Avoid making statements when there is not statistical support.

We removed that statement.

Fig 2F-H, Fig 3 (all except G and maybe F)
To facilitate comparisons among these genotypes, the data in these panels should be consolidated so that there is one graph of each type:
Brood size
Survival
UV sensitivity
Mutations/generation
Deletion sizes
(Possibly Indel type pie charts, although those could be deferred to the next figure.)
(It is ok if not all genotypes are represented in every graph type)
The narrative in the Results section can still consider subsets of the data sequentially as the picture is being built up, as in the current version.

The figures have been amended considerably to allow for a better comparison of the different genotypes.

Fig 3G
3G and 3 F could potentially stand alone as a figure by themselves (new Fig 4), or they could be the last 2 panels of the new revised Fig 3,
However it is done, that the heat map for the random in silico generated deletions should be the same size as the real data for better comparison. Also, the actual values in the +1 -1 position should be indicated below the graph for each genotype and random. Finally, some evidence regarding whether there is statistical support for microhomology should be provided. One simple way would be to use a Chi-square test to assess, for the +1 -1 position, whether the incidences of same vs different nucleotide in the real deletions observed for each genotype differ from those expected for random. (Based on the visual representation and low numbers for rev3 single mutant, there may not be statistical support for this genotype; if that turns out to be the case, the text should be revised appropriately.)

The figures have been amended to address the comments above.

The text has been revised to state “This class of deletions, with a narrow size range of 50-500bp, bears the typical signature of polymerase theta action upon DNA break repair: the occasional presence of template insertions, and an overrepresentation of micro-homology at the deletion junction (Fig. 3F,G)”, which to us relieves us from establishing statistical support for the individual genotypes which would indeed be impossible for a genotype that only has 15 cases.

P 10 L234-5. Change order of sentence to avoid semantic ambiguity: “”…repair, micro-homology at the deletion junction (G) and the occasional presence of…(F)”

This ambiguity has been addressed.

P 11 L253-5. This sentence is too definitive- need alternative wording that doesn’t overstate. E.g. need to consider/discuss the possibility that more lesions might form under these mutant conditions, and/or if this is considered unlikely, explain why.

While this reviewer rightfully made us aware of too strong wording I tend to disagree on this occasion but I can only call upon the principals of parsimony to rebut: we indeed cannot formally exclude the suggested alternative explanation (or any other) but it would require us to introduce a (at least to me) yet unknown phenomenon, while our interpretation is consistent with published genetic and biochemical data demonstrating that TLS polymerases can bypass lesions in an error-free manner. I feel that we have not overstated by using “argues” in the original version of the manuscript, but nevertheless have replaced it with “to us argues” to soften it.

P 11 L261. Section heading is too definitive- even though it is a reasonable explanation for the result, the section heading should state the finding, not the interpretation.

The section heading has now been changed to state the finding.

Discussion:
P 12 The discussion opens with the statement that TLS polymerases in yeast were found to “promote both induced and spontaneous mutagenesis”
HERE, the authors show that LACK of TLS polymerases apparently increases mutagenesis, implying that the TLS polymerases (as least as a cohort) INHIBIT mutagenesis. This apparent contrast should be addressed explicitly and head-on.

This has now been done.

P13 L308 -314. This is a model, with features that are consistent with the data. The authors need to more clearly distinguish between what is shown and models that are supported by the findings.
The data clearly show that the TLS polymerases are anti-clastogenic. They also show that Cs are overrepresented adjacent to deletion junctions. These data are consistent with a model in which: 1) breaks that give rise to the observed deletions tend to occur at or near the sites of damaged bases that cause replication blockage and 2) damaged Gs are the most common replication blockages that require TLS polymerases for their bypass.

The wording has been changed accordingly.

The discussion of amounts of spontaneous DSBs on P 14 completely ignores the possibility of elimination of cells with DSBs by apoptosis and/or use of HR to repair such breaks. These are relevant to the discussion, as both HR and DNA damage checkpoints are highly active in the germline.

We now include these options in the discussion sections.

I’m a bit confused by the last sentence of the discussion- hasn’t it already been argued that bypass using the TLSs in C. elegans is predominantly error free ( based on an increase in overall mutation frequency when they are absent)? If so, where is the trade-off?

We removed this indeed rather ambiguous statement.

We would like to very much thanks this (and also the other) reviewer(s) for taking the time to better our manuscript!
